# Maternal near miss among women admitted in major private hospitals in eastern Ethiopia: a retrospective study

**DOI:** 10.1186/s12884-021-03677-w

**Published:** 2021-03-05

**Authors:** Shegaw Geze Tenaw, Nega Assefa, Teshale Mulatu, Abera Kenay Tura

**Affiliations:** 1 Department of midwifery, College of Medicine and Health Sciences, Wolkite University, Wolkite, Ethiopia; 2 School of nursing and midwifery, College of Health and Medical Sciences, Haramaya University, P.O.B. 235, Harar, Ethiopia; 3 Department of obstetrics and gynecology, University Medical Centre Groningen, University of Groningen, Groningen, the Netherlands

**Keywords:** Maternal near miss, Private hospitals, Ethiopia, Maternal audit

## Abstract

**Background:**

Since maternal mortality is a rare event, maternal near miss has been used as a proxy indicator for measuring maternal health. Maternal near miss (MNM) refers to a woman who nearly died but survived of complications during pregnancy, childbirth or within 42 days of termination of pregnancy. Although study of MNM in Ethiopia is becoming common, it is limited to public facilities leaving private facilities aside. The objective of this study was to assess MNM among women admitted in major private hospitals in eastern Ethiopia.

**Methods:**

An institution based retrospective study was conducted from March 05 to 31, 2020 in two major private hospitals in Harar and Dire Dawa, eastern Ethiopia. The records of all women who were admitted during pregnancy, delivery or within 42 days of termination of pregnancy was reviewed for the presence of MNM criteria as per the sub-Saharan African MNM criteria. Descriptive analysis was done by computing proportion, ratio and means. Factors associated with MNM were assessed using binary logistic regression with adjusted odds ratio (aOR) along with its 95% confidence interval (CI).

**Results:**

Of 1214 pregnant or postpartum women receiving care between January 09, 2019 and February 08, 2020, 111 women developed life-threatening conditions: 108 MNM and 3 maternal deaths. In the same period, 1173 live births were registered, resulting in an MNM ratio of 92.1 per 1000 live births. Anemia in the index pregnancy (aOR: 5.03; 95%CI: 3.12–8.13), having chronic hypertension (aOR: 3.13; 95% CI: 1.57–6.26), no antenatal care (aOR: 3.04; 95% CI: 1.58–5.83), being > 35 years old (aOR: 2.29; 95%CI: 1.22–4.29), and previous cesarean section (aOR: 4.48; 95% CI: 2.67–7.53) were significantly associated with MNM.

**Conclusions:**

Close to a tenth of women admitted to major private hospitals in eastern Ethiopia developed MNM. Women with anemia, history of cesarean section, and old age should be prioritized for preventing and managing MNM. Strengthening antenatal care and early screening of chronic conditions including hypertension is essential for preventing MNM.

## Background

At the end of the Millennium Development Goals, ending maternal mortality remained the unfinished agenda and improving maternal health, well-being and survival remains central goal and investment priority of the Sustainable Development Goals [1]. Although maternal mortality decreased by 38% since 2000 in sub-Saharan Africa, 66% of the 295,000 maternal deaths in 2017 occurred in the region [2].

Globally, maternal mortality is used to monitor maternal health, quality of reproductive health care, and progress towards international goals [2, 3]. However, maternal death is a rare event in absolute terms at individual health facilities, including in high burden countries limiting their significance in generating essential lessons for improving maternal health [4]. In addition, maternal mortality figures, by definition, shows negative endpoint irrespective of interventions provided. These led to the emergence of the concept of maternal near miss (MNM), for evaluating the quality of maternal health care as a proxy indicator for measuring maternal health [4, 5].

According to the World Health Organization (WHO), MNM is defined as “a woman who nearly died but survived a complication that occurred during pregnancy, childbirth or within 42 days of termination of pregnancy” [5]. MNM could serve as a proxy indicator for maternal death and allows for more rapid assessment of maternal health care. It also serves as a surrogate to gain a better understanding of a set of conditions and preventable factors which contribute to maternal death [6]. Even though maternal mortality is considered to be one of the main indicators of maternal health, it shows only a small fraction of the maternal ill-health continuum and understanding the true burden of maternal ill-health requires studying near miss in addition to maternal deaths.

Depending on the definitions applied, for every woman in Ethiopia who dies from pregnancy related causes, 12 to 21 others experience near miss [7, 8]. In addition, MNM studies in low resource settings are subjected to under reporting because of low applicability of the MNM identification criteria [9–11]. So, a proposed sub-Saharan African MNM tool was developed through an international Delphi study [12]. In three rounds of Delphi among experts who used the existing WHO MNM tool in sub-Saharan Africa, the existing WHO MNM criteria were rated based on their feasibility with regard to diagnostic capabilities, presence of infrastructure and ability to diagnose the conditions. As such, the sub-Saharan African MNM tool containing 27 MNM indicators was developed. Details of the sub-Saharan African MNM tool and the Delphi study has been described elsewhere [12]. The applicability of the sub-Saharan African MNM tool has been tested in Ethiopia and Namibia [8, 13].

Although studying of MNM in Ethiopia is becoming common since the first study in 2012, almost all the studies are limited to public health institutions [8, 14–17]. Given the existence of variation in demographic, obstetrics and medical characteristics of women in public and private hospitals, and private facilities contributing significantly to care of women in pregnancy and childbirth [18, 19], existing studies failed to include the perspectives of private facilities and therefore our understanding of MNM is incomplete. Although a recent secondary analysis of the 2016 Ethiopian Emergency Obstetric and Newborn care estimated MNM, including in private facilities, the study failed to clearly indicate the burden separately for private facilities [20]. The objective of this study was to assess prevalence of MNM and associated factors among women admitted in major private hospitals in Harar and Dire Dawa Cities, eastern Ethiopia.

## Methods

### Study settings

The study was conducted in the obstetrics and gynecology units of two major private hospitals in Harar and Dire Dawa towns, eastern Ethiopia: Harar General Hospital and Bilal General Hospital. Harar General Hospital is a 33 bedded (five ICU) hospital serving for both referred and self-referred women, especially for a better off woman. During the study period, the unit was run by five consultants and six midwives. An estimated 900 deliveries occur per annum [21]. Bilal Hospital is a general 12 bedded (four ICU) hospital in Dire Dawa run by one consultant and seven midwives [22]. Both hospitals have one major operation theatre shared for all types of surgery. Unlike the public facilities, where all maternity services are free, majority of the women in these hospitals are from better off population and urban residents paying for all hospital services. The study was conducted from March 5 to 31, 2020.

### Study design and population

Institution based cross sectional study was conducted among women admitted in the two hospitals during pregnancy, childbirth or within 42 days of termination of pregnancy during the period of January 9, 2019 to January 08, 2020 and fulfilled the validated sub-Saharan African MNM tool [8, 12, 13]. The sub-Saharan African MNM criteria contains 27 indicators (including 19 from WHO MNM tool) grouped in to clinical, laboratory and management-based approaches. The tool has already been tested in two studies from Ethiopia [8] and Namibia [13] and has been found to be effective for MNM studies in low resource settings. Incomplete medical records with missing of important variables were excluded. The minimum sample size was calculated using the WHO recommendation for calculating prevalence of severe maternal outcomes divided by the number of women giving birth within a given time period [23]. Considering the existing maternal mortality ratio and the annual number of deliveries, a total of 1000 live births were sufficient to identify 100 women with severe maternal outcomes. But considering the overall low deliveries in private facilities, we included all women who were admitted during the study period.

### Data collection

Data were collected through review of all medical records using a standard checklist prepared for this purpose. Trained research assistants collected data on socio-demographic conditions of the woman, obstetrics history, pre-existing medical conditions, MNM events, underlying complications, and treatment received. Identification of MNM events was a two-step process. *First*, all medical records of women were screened for presence of any potentially life-threatening conditions (severe postpartum hemorrhage, severe pre-eclampsia, eclampsia, uterine rupture, severe complication of abortion and sepsis/ severe systemic infection), received critical interventions (use of blood products and laparotomy other than cesarean section) or admitted to the intensive care unit [5]. *Then*, women who developed life-threatening complications consisting MNM and maternal deaths according to the sub-Saharan Africa MNM criteria were identified. Details of the sub-Saharan Africa MNM criteria, and its development and validation are described elsewhere [8, 12]. Information regarding whether the near miss was present before arrival or developed during hospitalization was collected to determine quality of care or delays in reaching facilities. Data on total number of deliveries and live births occurring during the study period for each hospital was extracted from the monthly hospital reports. The dependent variable was MNM defined as presence of any of the sub-Saharan Africa MNM criteria [8]. Independent variables included demographic characteristics (residence, age), obstetrics history (parity, history of cesarean section, history of abortion, history of stillbirth, and ANC utilization), and pre-existing medical conditions (chronic hypertension, anemia).

### Data processing and analysis

Data were coded; double entered and cleaned using EpiData 3.1 and exported to SPSS 20 for analysis. Descriptive statistics of study participants and MNM indicators were analyzed. MNM ratio, severe maternal outcome ratio, mortality index (proportion of women who died from all sustained severe maternal outcomes, MD/MNM + MD*100) and MNM to mortality ratios were calculated [5]. In addition, hospital access indicators, such as the number of women with an MNM condition before arrival at the hospital, and number of women with near-miss who developed conditions in the hospital were also calculated. Continuous variables like age and parity were recorded to discrete: age (< 20, 20–34, and > 35), parity (nullipara, 1–2, and > 3). Bivariate logistic regression analysis was performed to see the association between each independent variable and MNM. Independent variables with 푝-value of ≤0.25 were selected for multiple logistic regression after checking for multi-collinearity using the Variance Inflated Factor (VIF) and standard error. Association was described using adjusted odds ratio along with 95% CI and *p*-value < 0.05 was considered as statistically significant.

## Results

**Fig. 1:**
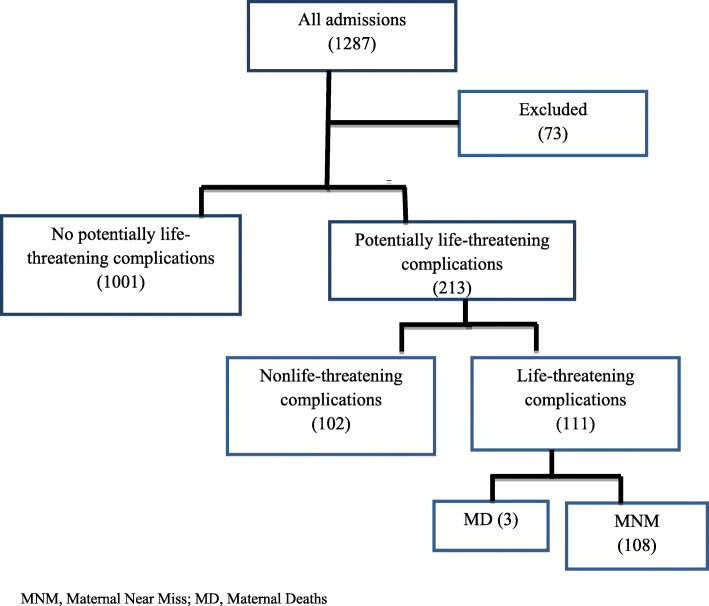
Flow chart of maternal near miss in major private hospitals in eastern Ethiopia, 2020. MNM, Maternal Near Miss; MD, Maternal Deaths

Of total 1287 admissions during pregnancy, childbirth, and within 42 days of termination of pregnancy, 1214 cases were included, after excluding 73 files which were incomplete or not accessible. During the same period, 1265 deliveries resulting in 1173 live births were registered in both hospitals. From these, 213 women were classified as having one or more of the potentially life-threatening conditions, and 111 developed life-threatening complications: 108 MNM and 3 maternal deaths (Fig. 1). This resulted in an MNM ratio of 92.1 per 1000 live births, 94.3 and 87.2 for Harar General Hospital and Bilal Hospital, respectively. One maternal death occurred in Harar General Hospital and the other two were in Bilal Hospital, corresponding with an overall institutional maternal mortality ratio of 256 per 100,000 live births.

### Characteristics of participants

The mean age of the participants was 26.4(+ 5.2) years, ranging from 17 to 45 years. Majority of the study participants were from urban (66.9%), admitted on working days (71%), self-referred (97.3%), received at least one ANC (92.5%) and had no history of abortion (79.9%) (Table 1).

**Table 1:** Socio-demographic and obstetric characteristics of women in major private hospitals in Eastern Ethiopia, 2020 (*n* = 1214)

Variable	Category	Frequency	(%)
Age in years	< 20	84	6.9
	20–34	1025	84.4
	≥35	105	8.7
Residence	Urban	812	66.9
	Rural	402	33.1
Days of admission	Working day	862	71.0
	Weekends	313	25.8
	Holidays	39	3.2
Referral status	Self-referral	1174	96.7
	Referred from other facility	40	3.3
Parity	0	233	19.2
	1–2	586	48.3
	3 or more	395	32.5
Received at least one antenatal care	Yes	1123	92.5
	No	91	7.5
History of still birth	Yes	85	7.0
	No	1129	93.0
History of abortion	Yes	244	20.1
	No	970	79.9
Previous cesarean section	Yes	167	13.8
	No	1047	86.2
Mode of delivery	Vaginal delivery	789	65.0
	Emergency cesarean section	330	27.2
	Elective cesarean section	85	7.0
	Abortion or laparotomy	10	0.8

### MNM indicators

As indicated in Table 2, the MNM ratio was 92.1 per 1000 live births and for every maternal death, there were 36 MNM cases. MNM ratio according to the WHO criteria was 40.9 per 1000 livebirths with lower MNM to mortality ratio (16:1) and higher morality index (5.9). The mortality index was 2.7%, and was highest among cases referred from other facilities (4.5%) compared to self-referrals (2.2%). High proportions (90; 81.1%) of MNM were already present on arrival or developed within 12 h of admission (Table 2).

**Table 2:** MNM indicators among women admitted in private hospitals in Eastern Ethiopia, 2020 [5]

Outcomes	Near miss indicators	
	SSA	WHO [5]
**1. All live births in the population under surveillance (n)**	**1173**	**1173**
**2. Severe maternal outcomes (SMO) cases (n)**	**111**	**51**
Maternal near miss (n)	108	48
Maternal death (n)	3	3
**3. Overall near miss indicators**		
Severe maternal outcome ratio (per 1000 live births)	94.6	43.5
Maternal near-miss ratio (per 1000 live births)	92.1	40.9
Maternal near-miss mortality ratio (MNM:MD)	36	16
Mortality index (%)	2.7	5.9
**4. Hospital access indicators**		
SMO cases presenting the organ dysfunction or maternal death within 12 h of hospital stay (SMO12) (number)	90	35
Proportion of SMO12 cases among all SMO cases	81.1	68.6
Proportion of SMO12 cases coming from other health facilities	21.1	14.3
SMO12 mortality index (%)	3.3	8.6
**5. Intra-hospital care**		
Intra-hospital SMO cases (number)	21	13
Intra-hospital SMO rate (per 1000 live births)	17.9	11.1

*SMO* severe maternal outcome, *MNM* maternal near miss, *MD* maternal death, *mortality index* number of maternal deaths divided by all severe maternal outcomes (maternal near miss and maternal deaths)

Transfusion of ≥2 units of blood (34.2%), sepsis or systemic infection (23.4%), and eclampsia (18%) were the three most prevalent MNM events (Table 3). Obstetric hemorrhage (54; 50%) and hypertensive disorders of pregnancy (30; 27.8%) were the major underlying causes of MNM while anemia (44; 40.7%) was the leading contributory cause of MNM (Table 4).

**Table 3:** Distribution of MNM events in women with life threatening complications in private hospitals, Eastern Ethiopia, 2020

Parameter	Frequency	Percent (%)
Maternal near miss(n)	108	
Maternal death (n)	3	
**Clinical criteria**		
Cyanosis	11	9.9
Gasping	4	3.6
Respiratory rate *>* 40 or *<* 6/min	6	5.4
Shock	19	17.1
Failure to form clots	1	0.9
Loss of consciousness lasting ≥12 h	2	1.8
Cardiac arrest	1	0.9
Stroke	1	0.9
Uncontrollable fit/total paralysis	2	1.8
Jaundice in the presence of pre-eclampsia	1	0.9
Eclampsia	20	18.0
Uterine rupture	9	8.1
Sepsis or severe systemic infection	26	23.4
Pulmonary edema	2	1.8
Severe abortion complications	7	6.3
Severe pre-eclampsia with ICU admission	12	10.8
**Any clinical criteria**	88	79.3
**Laboratory-based criteria**		
Oxygen saturation *<* 90% for *>* 60 min	15	13.5
Creatinine ≥300 μmol/l or ≥ 3.5 mg/dl	1	0.9
Acute thrombocytopenia (*<* 50,000 platelets/ml)	9	8.1
Loss of consciousness and ketoacids in urine	4	3.6
**Any Laboratory-based criteria**	27	24.3
**Management based criteria**		
Hysterectomy following infection or hemorrhage	9	8.1
Transfusion of ≥2 units of red blood cells	38	34.2
Intubation and ventilation for 60 min not related to anesthesia	7	6.3
Cardio-pulmonary resuscitation	6	5.4
**Any Management based criteria**	51	45.9
**Total severe maternal outcomes**^**a**^	166	

^a^Total exceeds total number of cases since some women have more than one inclusion criteria

**Table 4:** Underlying causes and contributing factors of life-threatening complications among women in major private hospitals, Eastern Ethiopia, 2020

	SMO	MNM	MD	MI
	n (%)	n (%)	n (%)	%
Over all	111	108	3	2.7
**Underlying causes**				
**Obstetric hemorrhage**	**55 (49.5)**	**54 (50)**	**1 (33.3)**	**1.8**
Abortion related	7 (6.3)	7 (6.5)	0 (0)	0.0
Ectopic pregnancy	4 (3.6)	4 (3.7)	0 (0)	0.0
Abruptio placenta	18 (16.2)	18 (16.7)	0 (0)	0.0
Placenta previa	7 (6.3)	7 (6.5)	0 (0)	0.0
Uterine rupture	9 (8.1)	8 (7.4)	1 (33.3)	11.1
Severe postpartum hemorrhage	13 (11.7)	12 (11.1)	1 (33.3)	7.7
**Hypertensive disorders**	**32 (28.8)**	**30 (27.8)**	**2 (66.7)**	**6.3**
Eclampsia	20 (18.0)	18 (16.7)	2 (66.7)	10
Preeclampsia	12 (10.8)	12 (11.1)	0 (0.0)	0.0
**Sepsis/severe systemic infection**	**26 (23.4)**	**26 (24.1)**	**0 (0.0)**	**0.0**
**Contributory causes**				
Anemia	46 (41.4)	44 (40.7)	2 (66.7)	4.3
Chronic hypertension	15 (13.5)	15 (13.9)	0 (0.0)	0.0
Heart disease	1 (0.9)	1 (0.9)	0 (0.0)	0.0

*MNM* maternal near miss, *MD* maternal death, *MI* mortality index, *SMO* severe maternal outcome

As shown in Table 5 coverage of key process indicators ranged from 69% for the use of oxytocin in severe postpartum hemorrhage to 80% for the use of magnesium sulphate in eclampsia. Mortality index was found to be highest among cases of uterine rupture (11.1%) and least among cases of severe postpartum hemorrhage (7.7%) (Table 5).

**Table 5:** Process and outcome indicators related with specific conditions among women with life-threatening complications in private hospitals of Eastern Ethiopia, 2020

**Indicator**	**Number**	**Percentage**
**1. Treatment of severe postpartum hemorrhage**		
Target population: women with severe PPH	13	
Oxytocin	9	69.2
Ergometrine	8	61.5
Misoprostol	10	76.9
Any uterotonics	13	
Artery ligation (uterine/hypogastric	1	7.7
Hysterectomy	4	30.8
Mortality	1	7.7
**2. Anticonvulsants for eclampsia**		
Target population: women with eclampsia	20	
Magnesium sulfate	16	80.0
Other anticonvulsants	4	20.0
Any anticonvulsant	20	100
Mortality	2	10.0
**3. Treatment for sepsis**		
Target population: women with sepsis	26	
Parenteral therapeutic antibiotics	20	76.9
**4. Ruptured uterus**		
Target population: women with ruptured uterus	9	
Hysterectomy	7	77.8
Mortality	1	11.1

*PPH* postpartum hemorrhage

### Factors associated with MNM

Age, ANC, history of previous cesarean section, chronic hypertension, and anemia in the index pregnancy were significantly associated with developing MNM (Table 6). Women > 35 years old were 2.29 times more likely to develop MNM compared to those 20–34 years old. The odds of developing MNM among women with no ANC were 3 compared to women with at least one ANC. In addition, MNM was more likely among women with previous CS, who had chronic hypertension and anemic (Table 6).

**Table 6:** Factors associated with MNM among women admitted in private hospitals in Eastern Ethiopia, 2020 (*n* = 1211)

Variable	MNM		95% confidence interval	
	Yes (***n*** = 108)	No (***n*** = 1103)	cOR	aOR
Age in years				
20–34	74 (7.2%)	950 (92.8%)	1.0	1.0
< 20	9 (10.7%)	75 (89.3%)	1.54 (0.74–3.20)	2.13 (0.86–5.27)
≥ 35	25 (24.3%)	78 (75.7%)	4.12 (2.47–6.84)	**2.29 (1.22–4.29)** ^*^
Residence				
Urban	65 (8%)	745 (92%)	1.0	1.0
Rural	43 (10.7%	358 (89.3%)	1.38 (0.92–2.07)	1.45 (0.92–2.29)
History of Stillbirth				
No	94 (8.3)	1033 (91.7%)	1.0	1.0
Yes	14 (16.7%)	70 (83.3%)	2.20 (1.19–4.05)	1.84 (0.93–3.64)
History of Abortion				
No	77 (8%)	891 (92%)	1.0	1.0
Yes	31 (12.8%)	212 (87.2%)	1.69 (1.09–2.64)	1.57 (0.96–2.57)
ANC utilization				
Yes	92 (8.2%)	1028 (91.8)	1.0	1.0
No	16 (17.6%)	75 (82.4%)	2.38 (1.33–4.26)	**3.04 (1.58–5.83)** ^**^
Parity				
1–2	35 (6.0%)	551 (94.0%)	1.0	1.0
0	18 (7.8%)	214 (92.2%)	1.32 (0.73–2.39)	1.76 (0.85–3.64)
≥ 3	55 (14%)	338 (86%)	2.66 (1.71–4.13)	1.58 (0.94–2.67)
Previous CS				
No	71 (6.8%)	974 (93.2%)	1.0	1.0
Yes	37 (22.3)	129 (77.7%)	3.94 (2.54–6.10)	**4.48 (2.67–7.53)** ^***^
Chronic Hypertension				
No	94 (8.2%)	1048 (91.8%)	1.0	1.0
Yes	14 (20.3%)	55 (79.7%)	2.84 (1.52–5.29)	**3.13 (1.57–6.26)** ^**^
Anemia in current pregnancy				
No	66 (6.3%)	989 (93.7%)	1.0	1.0
Yes	42 (26.9%)	114 (73.1%)	5.52 (3.58–8.51)	**5.03 (3.12–8.13)** ^***^

*MNM* maternal near miss, *ANC* antenatal care, *CS* cesarean section, *cOR* crude odds ratio, *aOR* adjusted odds ratio; ^*^ *p* = 0.010, ^**^ *p* = 0.001, ^***^ *p* = 0.000

## Discussion

We found that the MNM ratio in major private hospitals in eastern Ethiopia was 92.1 per 1000 live births (95% CI: 72–105). Majority of the MNM cases were already present on arrival or developed within 12 h of admission. MNM was more likely among women aged > 35 years, not have antenatal care, have history of cesarean section, anemic or have chronic hypertension.

Compared to previous studies which utilized the sub-Saharan Africa MNM tool, our finding is comparable with finding in Harar (80.2) [8], but higher than the study in public health facilities in Namibia (31.9) [13]. The variation might be due to differences in the study population or study settings (private vs. public). Compared to public facilities, where the Namibian study was conducted, women coming to private facilities are high risk population seeking specialized private care [24, 25]. In addition, as only women from high income population or with known risk factors are seeking private facilities, there might be under estimation of live births (denominator of MNM) among low risk population which may use public facilities or lower facilities like health center in our study.

As expected, the MNM according to the WHO MNM criteria (40.9 per 1000 livebirths) resulted in under estimating MNM compared to the sub-Saharan Africa MNM criteria (92.1 per 1000 live births). A similar under reporting of MNM in low resource settings by the WHO MNM criteria was previously reported [9–11]. The MNM ratio according to the WHO MNM criteria (40.9 per 1000 livebirths) is still higher compared to similar MNM studies in public health facilities in Ethiopia except a study by Woldeyes et al. [26] in Jimma, south western Ethiopia (50.4). A study conducted in two public facilities in the same location reported an MNM ratio of 17.2 per 1000 livebirths [8]. Our finding is, however, similar with MNM findings in Nigeria and Zanzibar, Tanzania [27, 28].

Our finding is much lower than the MNM ratio reported in a private tertiary hospital in Nigeria (262 per 1000 live births) [29] and the national estimates for Ethiopia (208 per 1000 live births) [20]. Given that majority of patients visiting a private tertiary maternity hospital in the Nigerian study would be high-risk population compared to our general hospitals, this difference is expected. In addition, since the Ethiopian study included all women with any complication as a maternal near miss, this variation is also not unexpected [20].

In line with previous studies in Ethiopia and other low-resource settings, large number of women had MNM on arrival or developed within 12 h of admission [17, 26]. This indicates marked delays in recognizing complications (*delay one*) or reaching the appropriate facilities (*delay two*) [5]. Given that majority of women were from urban areas and came to the facilities without referral, interventions to improve early recognition of complications are essential.

The MNM to mortality ratio of this study (36:1) is higher than findings from public hospitals in Harar (21:1 [8] and Jimma (5.8:1) [26]. This might reflect that women are receiving quality specialized care directly compared to the long chain of hierarchical care by different level of specialties before senior consultations in public facilities. This could also be characterized by referring out of critical patients to the nearby public referral hospitals, which might end up as ‘death on arrival’ elsewhere [30]. Although the World Health Organization recommends magnesium sulphate for all women with eclampsia [5], only 80% of cases with eclampsia received magnesium sulphate. Similarly, only 69% of women with severe PPH received oxytocin. This should be assessed whether it was due to lack of supplies or poor documentation.

Consistent with findings from Australia [31], Brazil, and the United Kingdom [32, 33], MNM was more likely among older women (≥35 years). Pregnancies in older women carries greater risk of hypertensive disorders of pregnancy, cesarean section or postpartum hemorrhage [34, 35] which are known risk factors for MNM. Women with no ANC were also more likely to develop MNM compared to women who had at least one. Similar association of no ANC and MNM has been previously reported in Ethiopia [36, 37], Brazil [38], and Nigeria [39]. This indicates the importance of ANC in identifying pregnancy complications and providing early treatment which gives the opportunity for early detections and treatments of complications thereby reducing near miss events.

Similar with previous findings in Ethiopia [14], Tanzania, and Brazil [38, 40], MNM was more likely among women who had history cesarean section. Women with prior cesarean section carry higher risk of uterine rupture, placenta previa or PPH in subsequent pregnancies [41]. In congruent with findings from studies in Ghana and Addis Ababa (Ethiopia), anemic women had higher risk of developing MNM [36, 42]. Nutritional intervention and iron supplementation for all women during pregnancy may help to prevent and improve anemia during pregnancy [43]. In addition, women with chronic hypertension were more likely to develop MNM than their counterparts, a finding previously reported in Ethiopia [36] and Nigeria [44]. This could be explained by the fact that chronic hypertension increases risk of severe pregnancy complications like super imposed preeclampsia and placental abruption thereby increasing odds of MNM [45].

We included all women admitted during pregnancy, childbirth or within 42 days of termination of pregnancy over 1 year and therefore not prone to sampling errors. However, we were unable to comment on the timeliness of treatments received and delays associated with management since the time between decision and actual treatments was rarely documented. In addition, majority of sociodemographic characteristics (income, educational status, partner’s status, and occupation) which affect treatment seeking or outcomes were not assessed since they are not routinely documented in medical records. In addition, lack of studies in private hospitals in general and which utilized the adapted sub-Saharan African MNM criteria in particular, made comparing our findings with others to be difficult.

## Conclusion

We found that the risk of MNM was significantly higher among women > 35 years, had no ANC, have history of CS, anemia in index pregnancy and chronic hypertension. Improving maternal and prenatal outcomes in private facilities should emphasize on women at risk of MNM: older, previous CS, anemic, no ANC, and chronic hypertension. Implementation of prenatal risk identification and prompt referral to the nearby hospitals are essential for reducing delays in provision of appropriate care. In addition, audit of appropriateness of treatments and delays in management of women in private hospitals is required if the goals for ending preventable maternal mortality is to be achieved.

## Data Availability

All data used for conclusion in this study are included in this article. Additional data are available from the corresponding author on reasonable request.

## Abbreviations

ANC: Antenatal Care
CS: Cesarean Section
ICU: Intensive Care Unit
MD: Maternal Death
MNM: Maternal Near Miss
PPH: Postpartum Hemorrhage
WHO: World Health Organization
